# Designing and implementing a communications strategy: lessons learnt from HIV and Sexual and Reproductive Health Research Programme Consortia

**DOI:** 10.1186/1478-4505-9-S1-S15

**Published:** 2011-06-16

**Authors:** Annabelle South

**Affiliations:** 1 Department of Infectious Disease Epidemiology, London School of Hygiene and Tropical Medicine, Keppel Street, London, WC1E 7HT, UK

## Abstract

In recent years there has been increasing recognition of the importance of strategic research communication. Health research organisations need to be able to communicate their research effectively to increase the probability that the findings influence policy and practice, and benefit those in greatest need. Many research funders are making communications a requirement of research funding. This paper reflects on the experience in developing and implementing communications strategies of several Research Programme Consortia funded by the Department for International Development.

Different research topics will require different communications approaches in order to be effective. This is reflected in the diversity of strategies employed by different research programmes. Strategic research communications designed to influence policy and practice require different skills and expertise from those required for carrying out research and writing it up for publication in academic journals. Therefore researchers involved in communicating research should be supported in this work. One of the ways in which research programme consortia have sought to do this is through convening workshops to develop the communications skills of researchers from partner organisations. These have proven invaluable. Another way of providing ongoing support to those involved in communicating research is through a Communications Community of Practice. Where this has been used it has proven a good way to support researchers both with ideas and resources, but also a strong sense of belonging to a community that shares a common concern with communication. Developing strong partnerships with research users, other research organisations, knowledge intermediaries and other stakeholders is vital for effective communication.

Embracing the challenges and opportunities presented by communicating research to influence policy practice is vital if research is to have maximum possible impact, and demonstrate its worth at a time when funding for health and development activities is at risk. Sharing lessons learnt in this process between research programmes is important to support this work.

## Introduction

In recent years there has been increasing recognition of the importance of strategic research communication. There is international consensus that health policy should be evidence-informed [1]. There is also growing realisation that carrying out good research, and publishing the results in academic journals, is not enough for research to influence policy and practice. The Ministerial Summit on Health Research, held in Mexico in November 2004, called on “All major stakeholders to strengthen or to establish activities to communicate, improve access to, and promote the use of reliable, relevant, unbiased, and timely health information” [1].

Research funders are increasingly making communications a requirement of research funding. The UK Department for International Development (DFID) played a pioneering role with its requirement that at least 10% of funding for Research Programme Consortia (RPC) should be spent on communication [2]. AusAID and DANIDA have both also introduced a similar requirement. (The recent change in government in the UK has led to DFID’s funding for research communication being rebranded as research uptake). A third of the marks for proposals for EU collaborative research funding are based on ”Potential impact through the development, dissemination and use of project results” [3], with research communications being key components of this. Other donors are using different mechanisms for encouraging research communications, such as: making it compulsory for research projects to have communications strategies; developing the capacity of researchers to communicate and policy makers to uptake research evidence; encouraging and funding knowledge intermediaries; and making the involvement of stakeholders from the start of the project compulsory [4].

Strategic research communications designed to influence policy and practice require different skills and expertise from those required for carrying out research and writing it up for publication in academic journals. Many researchers are not trained in these skills, and research organisations often lack communications capacity, as it has not traditionally been a requirement. The emphasis on communicating research has challenged individuals and organisations to develop new ways of doing things, and work with a new range of partners and communications professionals.

This paper reflects on the experience in developing and implementing a communications strategy of several RPC funded by DFID. RPC are collaborative international research consortia that specialise in particular research themes, and “Aim [...] to generate new policy-relevant knowledge that will help developing countries, the wider development community and DFID to eradicate world poverty, including meeting the MDGs by 2015” [5]. The consortia include organisations from both developed and developing countries, and often include both academic and civil society organisations, as well as government institutions. Funding is for 5-6 years, and is for a programme of work on agreed themes, rather than a specific research project. Through this funding mechanism DFID encourage communication of research and capacity building, in addition to high quality research.

Health researchers and research organisations need to be able to communicate their research effectively to increase the probability that the findings influence policy and practice, and benefit those in greatest need. By sharing the lessons learnt in this process by several RPC working on HIV and Sexual and Reproductive Health (SRH), it is hoped that this paper will help other research projects and programmes facing similar issues.

## Discussion

### Developing communications strategies

Developing communications strategies, outlining the objectives, audiences and communication methods, from the planning stage of the research is important to allow relationships with audiences to be built over the course of the project. Different topics will require different approaches in order to be effective, so it is not possible to ‘cut and paste’ strategies from one project to another. The foci of the communications strategies for different RPC are very different. For example, the Evidence for Action RPC, which works on HIV treatment and care systems, focused its communications strategy on national and international policymakers and programme managers. Whereas Realising Rights, which dealt with hotly contested issues around sexual and reproductive health rights, focused on the media and parliamentarians in order to try and foster a more open and positive attitude to the controversial issues it was investigating.

The process of developing a communications strategy can be a useful opportunity for developing the communications capacity of research partners. For example, Evidence for Action held a workshop bringing together the partners to look at how to develop a strategy, and strengthen capacity in some key communications skills. It was facilitated by external experts and had input from members of the consortium with expertise in specific communications areas. Participants were introduced to the RAPID framework for understanding how research can influence policy [6]. They carried out exercises from the RAPID toolkits on policy impact and successful communication to identify the audience; the desired changes; forces for and against change; the message and the messenger [7,8]. The draft strategies were then taken back to partner organisations for consultation and revision before being finalised. This process enabled country-specific strategies to be developed, and strengthened the capacity of communications leads to plan strategic communications. It has resulted in strong buy-in from communications leads, who, in some cases, have acted as advocates within their organisation for communicating research.

### Implementing communications strategies

#### Capacity strengthening

Carrying out effective communications with non-academic audiences is complex, and requires different skills to those gained in traditional research training. Therefore, where researchers are expected communicate as well as carry out research, it is important to put in place support mechanisms, such as training and access to resources and expertise. One of the ways in which the RPC have sought to do this is through convening workshops to develop the communications skills of researchers from partner organisations. Evidence for Action, SRH & HIV RPC and Realising Rights RPC have all held communications workshops, which were invaluable for supporting the communications work of the consortia.

#### Human resources for research communications

One of the biggest challenges the RPC have faced in implementing the communications strategy is the limited time and human resources for carrying out communications activities. Many partner communication leads are primarily researchers, whose main responsibility is to actually carry out the research. They therefore have limited time to do communications activities, despite strong commitment to and enthusiasm for communications. Many RPC have found it crucial to have a person with communications skills who is championing communications within the consortium, coordinating the overall strategy and supporting partners to implement the communications strategy. Evidence for Action have also found it useful to have specific individuals from each partner organisation who are responsible for, and feel ownership of, communicating the research.

#### Community of practice

As a way of providing ongoing support for communication leads in partner organisations, Evidence for Action launched a Communications Community of Practice. This is a forum for communications leads to share experience, ideas and support. The Community of Practice meets regularly and tends to favour teleconferences over email and the internet for its communications. The communication workshops allowed people a chance to get to know each other in person. This has facilitated idea sharing and openness in the Community of Practice that would have been harder to build without the face-to-face start. After the Community of Practice had been established for 18 months, an evaluation was carried out to assess its relevance, importance and effectiveness to members, as well as to generate feedback on how to improve it. This was done through a survey of members. The evaluation found that members considered the Community of Practice a useful means of support, allowed them to make use of the expertise available within the consortium, provided inspiration and offered a sense of community. It was agreed to continue the Community of Practice through teleconferences and occasional workshops, while providing more access to resources via the Community of Practice intranet area and emails.

To be the only individual in an organisation who is involved in (or perhaps even values) communication can be an isolating experience. The community of practice has therefore been a good way to support the communications leads both with ideas and resources, but also a strong sense of belonging to a community that shares a common concern with communication. 5 out of 8 members agreed that feeling valued as a result of the comradeship of the community of practice was one of their motives for participating in it, and 6 out of 7 reported a strong sense of belonging to a community that has a common concern.

#### Importance of partnerships

A key recurring theme in the lessons from RPC communications work has been the importance of partnerships. The Evidence for Action Communications Community of Practice is an example of consortium partners benefiting from the experience of other members of the consortia. Partnerships with research users have also been crucial to effectively communicating the research. For example, in Uganda, Evidence for Action partners Medical Research Council/Uganda Virus Research Institute (MRC/UVRI) have been working in partnership with the Ministry of Health, with the Ministry of Health identifying a question of interest (can peripheral health services effectively and safely deliver ART?), and asking MRC/UVRI to work with it to find answers. We hope that this partnership from the start of research will allow the findings to be rapidly adopted. Walter et al. have found that “partnerships are most effective when research users are involved in all stages of the research process, rather than simply being co-opted during dissemination” [9]. Partnerships with media organisations and knowledge intermediaries have also been important for several RPC. For example, Realising Rights have worked in partnership with Panos to link research and media more effectively. Partnerships between the different RPC have also been useful, with four DFID-funded RPC on HIV and SRH (ABBA, Evidence for Action, Realising Rights and SRH & HIV) working together to examine the research to policy interface, and collaborating on a joint conference held in May 2010 [10]. This has enabled the different RPC to learn from each other’s experience, pool resources and reach wider audiences.

#### Financial resources for communications

Taking communications seriously requires dedicating financial resources to it. The DFID rule that at least 10% of the overall budget should be spent on communications has been important in ensuring that communications is taken seriously by all RPC, and enabling activities that are not cheap (such as communications workshops, buying in external expertise, and employing a dedicated communications manager, and holding major events) to happen. A review of DFID’s policy on communication for RPCs concluded that DFID’s 10% rule on research communication, and accompanying support, “Has clearly had a significant positive impact on communication activities within the RPC[s] themselves” [11].

## Conclusions

Effectively communicating research is essential if the full benefit of the research is to be realised in policy and practice. This is reflected by the growing requirement from donors that research projects carry out communications work. Effective research communications in the context of a consortium requires both financial and human resources for developing strategies; building the communications capacity of individuals and organisations; coordinating and supporting those individuals; as well as for communications activities and products themselves. The DFID requirement that at least 10% of the overall research budget should be spent on communications has helped to facilitate this within the RPC. However, there still remain competing pressures on the time of researchers which need to be taken into account when developing research and communications plans. Partnerships are crucial to effective communications, and the emphasis on getting research into policy and practice has already led to academic organisations engaging with different types of partners to those they traditionally work with. This offers new opportunities as well as new challenges. Embracing the challenges and opportunities presented by communicating research to influence policy practice is vital if research is to have maximum possible impact, and demonstrate its worth at a time when funding for health and development activities is at risk.

## Abbreviations

ABBA: Addresssing the Balance and Burden in HIV/AIDS Research Programme Consortium; AusAID: Australian Agency for International Development; DANIDA: Danish International Development Agency; DFID: Department for International Development; EU: European Union; HIV: Human Immunodeficiency Virus; MDGs: Millennium Development Goals; MRC/UVRI: Medical Research Council / Uganda Virus Research Institute; RPC: Research Programme Consortium; SRH: Sexual and Reproductive Health; UK: United Kingdom

## Competing interests

This paper critically reflects on a research project in which the author was involved.

## Author’s contributions

Single author.

## Author’s information

AS is the Communications Manager for Evidence for Action RPC.
